# What is the elevator pitch for open access?

**DOI:** 10.1002/nop2.74

**Published:** 2016-11-24

**Authors:** Roger Watson

**Affiliations:** ^1^ Nursing Open

A recent article by Leetaru (2016) in *Forbes* asked why academics had not embraced open access. After all, according to Leetaru, the academic community had been at the forefront, over the past two decades, of populating, using and promoting the spread of the Internet. The Internet, of course, is the medium for open access, yet it appears that open access regarding research findings is still not entirely accepted. This is despite the fact that academics want to share their research findings. The barriers may not, of course, rest entirely with the academics. The predominant model of academic publishing takes place by the traditional route of free submission of manuscripts and publication of articles by refereed journals. The articles are then read on a pay to view basis, mainly through collective agreements by, for example, university libraries to which employees and students have access. Rarely, individuals pay to view articles.

Recent years—approximately a decade or so—have seen the rise of the open access movement. This is a broad movement which includes mavericks or folk heroes—depending on your perspective—who have ‘broken ranks’ and made their own work available freely, ignoring the copyright restrictions of the major publishers. Others have made vast amounts of literature—their own and others—available open access. The arguments for this usually revolve around the nature of research being publicly funded and the ‘excessive profits’ of the major publishing houses (Watson, 2015). This is not the place to rehearse the arguments around the virtues of open access and the purported evils of the academic publishing industry. However, it is clear that the academic publishers have responded—along with many criminals in the shape of the predatory publishers (Pickler et al., 2015). The main academic publishing houses have responded in three ways:

1. Providing pay to publish open access options for articles accepted by the traditional route (the ‘gold route’)
2. Developing pay to publish open access online journals (also ‘gold route’)
3. Agreeing to allow final accepted manuscripts to be available on repositories, with an embargo period (the ‘green route’).

Options 1 and 2 above cost money in the form of an APC (article processing charge) and these can be expensive. They are expensive to offset the profits publishers may have made from pay to view. To obviate ‘double dipping’ whereby the publishers make money from selling open access on articles also available pay to view, major publishers have agreed to publish additional copy at no additional charge, to compensate. Option 3, the ‘green route’ is the only option free to the author and the reader but requires institutions to maintain repositories. The main driver for open access is making research outcomes more widely available and for academics there is also evidence—probably for selected outputs—that it may increase citations (Moed, 2012) to articles made available open access.

Given the cost of open access and the availability of the green route, why would an author pay an APC? As the elevator reaches its destination and you have to explain this to an author, what would you say? According to Mind Tools (undated) there are four key aspects of a—so‐called—elevator ‘pitch’:

- Identify your goal before trying to pitch under three points
- Explain what you do
- Communicate your USP
- Engage with a question

If ‘tasked’ with an elevator pitch on open access I think my response would be:

- My goal is to convince an author that publishing open access by the pay to publish route is worthwhile. Adapting the above slightly my three points, would be:
- We provide gold route open access options for authors to make their work freely available on the Internet.
- Open access by the gold route is the only way that your material can become instantly and freely available to read from the point of publication
- Open access can improve the impact of your research and citations to your articles; can you afford not to consider open access for your next publication?
